# Premature Progesterone Rise Is Associated with Higher Cumulative Live Birth Rate with Freeze-All Strategy

**DOI:** 10.3390/jcm13123439

**Published:** 2024-06-12

**Authors:** Yu Wang, Ming-Jer Chen, Hwa-Fen Guu, Ya-Fang Chen, Hsiao-Fan Kung, Jui-Chun Chang, Li-Yu Chen, Shih-Ting Chuan, Yu-Chiao Yi

**Affiliations:** 1 Division of Reproductive Endocrinology and Infertility, Department of Obstetrics, Gynecology & Women’s Health, Taichung Veterans General Hospital, Taichung 40764, Taiwan; adosgreenshow123@gmail.com (Y.W.); mingjerchen@gmail.com (M.-J.C.); hfku@vghtc.gov.tw (H.-F.G.); sylvan159x@yahoo.com.tw (Y.-F.C.); eminem4389@gmail.com (H.-F.K.); r.juichun@gmail.com (J.-C.C.); liyu0724@hotmail.com (L.-Y.C.); smile7966@hotmail.com (S.-T.C.); 2 School of Medicine, Department of Obstetrics and Gynecology, National Yang Ming Chiao Tung University, Taipei 11221, Taiwan

**Keywords:** anti-Mullerian hormone, assisted reproductive technology, embryo transfer, in vitro fertilization (IVF), pregnancy rate, progesterone

## Abstract

**Background/Objectives:** This paper undertakes an investigation into the implications of premature progesterone rise (PPR) on pregnancy outcomes in freeze-all strategy cycles. **Methods:** A retrospective cohort study encompassing 675 IVF/ICSI cycles using a freeze-all strategy was enrolled. The cycles were categorized into two groups based on serum progesterone levels at the time of hCG administration: 526 cycles had levels below 1.5 ng/mL, while 149 cycles had levels equal to or above 1.5 ng/mL. **Results:** The findings revealed a significantly higher number of mature follicles and retrieved oocytes in patients with PPR across all AMH categories. Multiple analyses revealed factors influencing PPR, including the duration of induction and the number of retrieved oocytes. Within the same oocyte retrieval number group, patients with PPR demonstrated non-inferior pregnancy outcomes compared to non-PPR patients. Upon adjustment for age, AMH, and total follicle-stimulating hormone (FSH) dosage, PPR maintained a positive correlation with the cumulative live birth rate (LBR). **Conclusions:** The study showed that PPR correlates with an increase in retrieved oocytes while maintaining similar embryo quality and oocyte retrieval rates and results in a higher cumulative LBR.

## 1. Introduction

Since the first report of premature progesterone rise (PPR) during the late follicular phase in 1991, there has been considerable interest in the past 30 years [1]. PPR is generally defined as serum progesterone ≥ 1.2 ng/mL or ≥1.5 ng/mL on the day of the hCG trigger in controlled ovarian stimulation cycles [2,3,4]. This phenomenon is relatively common in in vitro fertilization (IVF) and intracytoplasmic sperm injection (ICSI) procedures, with an incidence rate of approximately 17–29% [5]. The risk factors for PPR included the use of daily follicle-stimulating hormone (FSH), the number of retrieved oocytes, peak estradiol level, type of stimulation protocol, and duration of the induction cycle [2,6,7,8].

It is well established that elevated progesterone levels can lead to gene dysregulation in the expression profiles of endometrial components [9], change the date of the implantation window, and affect endometrial receptivity [10,11,12].

Bosch et al. concluded that progesterone levels ≥ 1.5 ng/mL were linked to a lower ongoing pregnancy rate in a retrospective study [6]. In this study, the cumulative live birth rate per oocyte retrieval cycle was also adversely impacted. Therefore, some clinicians prefer to reduce the duration of induction when increased serum progesterone is observed during ovarian stimulation. Fortunately, this impact of PPR on endometrium receptivity can be solved in freeze–thaw cycles.

Besides the shift in the implantation window, whether the quality of embryos and pregnancy outcomes are affected by PPR is still a matter of debate. Several studies reported that PPR does not affect embryo quality [13,14,15], nor the success rate of frozen-thawed transfers [16]. Conversely, some studies have reported a higher number of retrieved oocytes but a lower rate of high-quality embryos and cumulative live birth rates [17,18].

Although the effects of PPR on endometrial receptivity have become much clearer recently [19], its impact on embryo quality is still far from settled [20]. The freeze-all strategy is more common these days, and the adverse effect of PPR on endometrium can be solved in frozen-thawed cycles, so the concept that PPR adversely affects pregnancy outcomes has been challenged in the freeze-all era. However, several studies revealed PPR may affect pregnancy outcomes, even in freeze–thaw cycles, and it is still questionable whether PPR affects the cumulative live birth rate with the frozen-all strategy [18,21]. Our study evaluates the outcomes of patients with PPR who underwent the freeze-all strategy, which excludes the impact of high progesterone on the endometrium during fresh embryo transfers (ET). By focusing on the freeze-all strategy, we aim to better understand the effects of PPR on cumulative pregnancy rates in FET cycles. This study offers valuable insights into the impact of PPR on pregnancy outcomes for each ovarian stimulation cycle.

## 2. Materials and Methods

### 2.1. Subjects

This retrospective cohort study was carried out at Taichung Veterans General Hospital in Taiwan. Women who underwent IVF and/ or ICSI with the GnRH antagonist protocol between 1 January 2016, and 31 December 2021, were enrolled. Exclusion criteria were as follows: (1) patients diagnosed with adenomyosis via two-dimensional transvaginal sonography (TVS) within the past 12 months, (2) patients receiving donated oocytes, (3) patients receiving fresh embryo transfers, (4) patients with immune diseases or metabolic diseases, (5) patients with embryos transferred involving mixed stimulation cycles. Serum progesterone was measured using an Abbott Progesterone Reagent Kit performed by the Chemiluminescent Microparticle Immunoassay (CMIA).

Based on the serum progesterone levels on the day of hCG administration, patients were divided into two groups: those with progesterone levels below 1.5 ng/mL and those with progesterone levels equal to or exceeding 1.5 ng/mL. Based on study by Bu et al. [21], patients were categorized into three groups according to their oocytes retrieved number: poor ovarian responder (retrieved oocytes ≤ 5), intermediate ovarian responder (retrieved oocytes between 6 and 19), and high ovarian responder (retrieved oocytes ≥ 20). We analyzed patients in all groups who had completed their treatment cycles, either by achieving a live birth or by ultimately not succeeding after transferring all their embryos.

### 2.2. Controlled Ovarian Hyperstimulation Protocol

The patients were administered the GnRH antagonist cetrorelix acetate (Cetrotide, 0.25 mg/d SC; Merck Serono, Darmstadt, Germany). This treatment was initiated flexibly between stimulation days 5 and 7, with ultrasound monitoring occurring 5 days after starting controlled ovarian hyperstimulation with gonadotropins.

Gonadotropin types and doses were tailored for each participant based on various factors, including age, body mass index, anti-Müllerian hormone (AMH) levels, follicle-stimulating hormone (FSH) and luteinizing hormone (LH) levels, antral follicle count on cycle days 2 to 3, and previous ovarian stimulation response. Adjustments to the doses were made according to the ovarian response, as monitored through folliculometry and serum estradiol level tests.

When two or more follicles achieved a mean diameter of 18 mm, the ovarian trigger was scheduled. Patients received an injection of 0.2 mg GnRH agonist (Decapeptyl; Ferring Co., Kiel, Germany) and 250 mcg recombinant hCG (Ovidrel; Merck Serono, Germany). The dose of hCG could be adjusted based on the risk of OHSS. Oocyte retrieval was carried out 35 to 36 h post-trigger.

In the freeze–thaw cycle, patients were placed on an artificial hormone replacement regimen. The replacement regimen (Estradiol valerate 2 mg, Synmosa Co., Taiwan) involved a step-up dosing strategy, starting with 4–8 mg per day for 5 days, increasing to 6–12 mg per day for the next 5 days. This was followed by twice-daily vaginal progesterone (8% Crinone; Merck Serono, Germany) at 90 mg, in addition to 12 mg of estradiol valerate once the endometrial thickness exceeded 8 mm as assessed by ultrasound. Luteal support was provided and continued until the 10th week of gestation if pregnancy was confirmed.

Additionally, 0.1 mg of Decapeptyl (Ferring Co., Germany) was administered on the 6th day after initiating luteal support. Embryo transfers (ET) were performed on day 2, day 3, or day 5 of culture.

### 2.3. Outcome Measures

Serum βhCG was tested on day 14 of embryonic age, and levels greater than 5 mIU/mL were considered indicative of biochemical pregnancy if no gestational sac was later identified. The live birth rate (LBR) was defined as the percentage of cases resulting in a live newborn. The cumulative live birth rate per retrieval cycle was calculated as the percentage of cycles resulting in at least one live newborn. These outcomes were tracked until 31 May 2022.

### 2.4. Editorial Board Members and Editors

The study protocol was approved by the Ethics Committee of Taichung Veterans General Hospital under approval number CE2241 and adhered to relevant ethical guidelines. The Institutional Review Board of Taichung Veterans Hospital granted a waiver for documenting informed consent, as the research posed no more than minimal risk to the subjects and involved only data review. 

### 2.5. Statistical Analyses

Statistical analyses were conducted using IBM SPSS version 22.0. Patient characteristics, AMH levels, oocyte retrieval numbers, and pregnancy outcomes were examined using two-tailed *t*-tests and Chi-square tests. A *p*-value of less than 0.05 was considered statistically significant.

## 3. Results

In total, 2561 cycles were reviewed, and 675 cycles were enrolled, including 526 cycles with serum progesterone < 1.5 ng/mL and 149 cycles with progesterone ≥ 1.5 ng/mL on the day of hCG administration. The baseline demographic analysis is shown in Table 1. There were no significant differences in age, total FSH dosage, FSH/LH dosage ratio, good embryos rate and blastocyst formation rate between patients with and without PPR. However, significant differences were found in AMH, induction duration, the number of follicles greater than 14 mm, estradiol on the day of hCG administration, and the number of oocytes retrieved.

As shown in Table 1, patients with PPR demonstrated significantly higher AMH levels and a greater number of oocytes. To clarify the relationship between increased oocyte number, AMH, and PPR, we analyzed the univariate and multivariate logistic regression analyses for serum progesterone ≥ 1.5 ng/mL. Table 2 illustrates that age, AMH, induction duration, number of follicles and oocytes, and estradiol levels on the day of hCG administration all have an impact on PPR. Serum progesterone appeared to be unaffected by the administered FSH/LH ratio, good embryos rate, and good blastocytes rate, and oocyte retrieval rates. In multivariate logistic regression analysis, when consideration of age, AMH, induction duration, and number of retrieved oocytes, it is the induction duration and number of retrieved oocytes but not age and AMH that showed significant effects on PPR.

The POSEIDON criteria classify patients into four groups based on ovarian reserve, age, and response to ovarian stimulation. The cutoff values for classifying patients into Groups 1 and 2 versus Groups 3 and 4 are an antral follicle count (AFC) of less than 5 and an AMH level of less than 1.2 ng/mL [22]. Patients with AMH less than 1.2 ng/mL was believed to expect a low number of oocytes according to the Poseidon criteria. AMH greater than 3.36 ng/mL was otherwise believed to have an increased risk of ovarian hyperstimulation syndrome [23]. In stratification analysis according to AMH, both groups of AMH < 1.2 ng/mL and AMH > 3.36 ng/mL revealed no significant differences between PPR patients and non-PPR patients in age, AMH, blastocyst formation rate and oocyte retrieval rate. However, the results showed a significant increase in retrieved oocytes number in PPR patients in both groups. (AMH < 1.2 ng/mL 4 oocytes versus 8 oocytes, *p* = 0.002; AMH < 1.2 ng/mL 9 oocytes versus 14 oocytes, *p* < 0.001; AMH > 3.36 ng/mL 21 oocytes versus 24 oocytes, *p* = 0.003) (Table 3).

Our results aim to evaluate the effect of PPR on pregnancy outcomes, including first FET live birth rates and cumulative LBR with the freeze-all strategy. As demonstrated in Table 4, a significantly higher cumulative LBR was found in patients with PPR (59.3% versus 71.9%, *p* = 0.002). Upon stratification analysis according to the number of retrieved oocytes, no significant differences were found between patients with and without PPR among poor ovarian responders, intermediate ovarian responders, or high ovarian responders.

To further explore the effect of serum progesterone level on trigger day, we divided patients into four groups according to the different progesterone quartiles in this study. The first quartile was patients with progesterone < 0.5 ng/mL; the second quartile was patients with progesterone greater than 0.5 and lower than 0.72 ng/mL; the third quartile was patients with progesterone greater than 0.72 and lower than 1 ng/mL; and the fourth quartile was patients with progesterone greater than 1 ng/mL. As illustrated in Table 5, patients in the highest progesterone group exhibited significantly higher first-FET live birth rates compared to the lowest progesterone group, as well as significantly higher cumulative live birth rates than all other lower progesterone groups. The lowest progesterone demonstrated significantly worse pregnancy outcomes not only in cumulative LBR but also in first FET LBR.

In addition, we performed a univariate and multivariate logistic regression analysis for cumulative LBR. The results are presented in Table 6. After accounting for age, AMH, and total dose of FSH, progesterone over serum progesterone ≥ 1.5 ng/mL still had a significant impact on the cumulative LBR.

## 4. Discussion

The impact of PPR on IVF outcomes has been a subject of concern for a long time, with conflicting results from different studies. Many studies revealed PPR may shift the implantation window in the fresh ET cycle and adversely affect the outcome of pregnancy [12,24,25]. However, some studies have concluded that there is no impact on pregnancy outcomes in FET cycles [3,14,16,26]. In contrast to the negative endometrial effect, the impacts of PPR on oocytes or embryo quality are still debatable. While some studies reported negative impact on embryo quality [18,27,28], several studies concluded that PPR does not have an impact on embryo quality [13,14,15,29]. In this study, we found that PPR was not associated with oocyte retrieval rates, good embryonic rates, or blastocyst formation rates. However, patients with PPR have significantly more retrieved oocytes even after adjustment for age, AMH, and induction days. The results confirm that PPR does not have an adverse effect on embryo quality but does increase the number of oocytes and cumulative LBR.

According to the two-cell, two-gonadotropin theory, theca cells produce progesterone, which then enters the granulosa cells to complete biosynthesis and be converted into 17β-estradiol [30]. In the process of ovarian stimulation with continuously elevated FSH levels but a lack of sufficient LH to support the activity of 17-alpha-hydroxylase, the increased amount of precursor steroids exceeds the ability of the ovary to transform them into estrogen. The delay in transformation results in a systemic elevation of serum progesterone. This model explains why FSH stimulation increases serum progesterone levels prior to ovulation [24]. Progesterone production during the follicular phase is contributed not only by the ovary but also by the adrenal gland. Increased estradiol concentration in stimulated cycles increases ACTH concentrations and stimulates progesterone production from the adrenals [31]. However, the precise mechanism behind progesterone elevation at the end of the follicular phase remains unclear.

From Table 1 and Table 2, significant positive correlations were found between PPR and younger age, higher AMH, increased number of oocytes, higher serum estrogen level on the day of the hCG trigger, and longer induction duration in our study. Furthermore, PPR appeared to not be associated with the FSH/LH ratio, good embryo rate, and oocyte retrieval rate. In the multivariate model, induction duration and oocyte number seem to be the dominant factors for PPR. Consistent with our results, Bosch et al. [6] reported that factors such as daily FSH dosage, the number of oocytes, and serum estradiol levels on the day of hCG administration are associated with PPR. A retrospective study analyzing over 10,000 cycles showed that PPR conferred the lowest risk when the total dosage of the LH/FSH ratio was between 0.3 and 0.6. This study further defined this range as a ‘sweet spot’ [32]. However, our results revealed that the LH/FSH ratio was not associated with the risk of PPR.

Kyrou et al. demonstrated that the size of the follicle was significantly associated with increased intrafollicular progesterone concentrations, and patients with more oocytes were expected to have higher serum progesterone [33]. Many studies also revealed that PPR was significantly associated with a higher number of retrieved oocytes [5,21,26,27,29]. A systematic review reported that the increase in the number of oocytes ranged from 1.9 to 3.1 oocytes in patients with PPR [5]. Neves et al. similarly observed that patients with PPR had significantly more oocytes retrieved, with an average of 17.67 ± 8.86 compared to 12.70 ± 7.00 (*p* < 0.001) [27]. Shimon et al. [26] reported that women with PCOS experiencing premature luteinization had a higher number of both retrieved and mature oocytes, and their clinical pregnancy rates were similar to those of patients without PPR. Consistent with the above studies, our results showed that patients with PPR had a significantly higher number of retrieved oocytes compared to those women with similar ovarian reserves. Werner et al. reported that high responders have a higher risk of PPR [32]. In our study, not only were high responders at risk of PPR, but even those women who were considered poor responders, i.e., patients with low ovarian reserve, were also at risk of PPR when they responded to ovarian hyperstimulation better than their cohort.

When it came to embryo quality, Neves et al. revealed in a study of IVF cycles with preimplantation genetic testing for aneuploidies (PGT-A) that patients with PPR had significantly higher number of euploids and a higher number of oocytes retrieved [27]. However, the euploid rate, blastocyte formation rate, first FET LBR and cumulative LBR were not significantly different. Similarly, Hill et al. reported that embryo stage and embryo quality were similar between the two groups [34]. Singh et al. [17] reported a non-inferior cleavage and fertilization rate in PPR patients. A meta-analysis study that included more than 60,000 donated oocyte cycles emphasized that there is no negative association between oocyte quality and PPR [5]. Contradictory findings on the association between progesterone levels and top quality embryo (TQE) are also reported. Huang et al. analyzed 4236 fresh ET cycles and reported that top-quality blastocyst formation rates were negatively associated with PPR [28]. Simon et al. also reported PPR with serum progesterone greater than 1.1 ng/mL, as the cutoff value was associated with lower TQE in the GnRH antagonist protocol [18]. Vanni et al. revealed that increasing progesterone is associated with lower rates of TQE [35]. Contrary to what has been reported by previous studies, recent studies reported by Hernandez-Nieto et al. and Pardinas et al. indicated that TQE was not significantly different between patients with and without PPR [36,37]. In our study, the good embryo rate and blastocyst formation rate were not significantly different between women with and without PPR, and the first FET LBR were similar among women with the second, third or fourth quartile of serum progesterone level. Patients with the same range of oocytes also appeared to have similar pregnancy outcomes, independent of PPR. These results indicated that the quality of the embryo has not been negatively affected by PPR.

For patients, the cumulative LBR is more valuable than the result of a single ET. There is a strong association between the number of oocytes and LBR [38]. Zhiqin et al. [21] reported that patients with elevated progesterone levels had significantly higher numbers of retrieved oocytes but a lower cumulative LBR. However, their report did not address the rate of fresh ET cycles that may be affected by PPR. Furthermore, an impaired cumulative pregnancy rate was observed after grouping based on the retrieved oocytes number, which seems to be an important factor affecting the cumulative pregnancy rate and was significantly associated with PPR in our study. To exclude the detrimental effect of PPR on the endometrium, we excluded women who received fresh ET, and our study showed that PPR had no negative impact on pregnancy outcomes in freeze-all cycles; conversely, women with PPR did have better outcomes in cumulative LBR. Taken together, these results suggest that the relationship between PPR and increased cumulative LBR is due to an increased number of oocytes, and the quality of oocytes is not inferior to that of non-PPR patients. Further multivariate logistic regression analysis also showed the same significant results. The results revealed that even after considering the effects of age, AMH, and dosage of FSH, patients with PPR had a higher cumulative LBR.

The study was limited in several ways. First, this was a retrospective study with a limited number of patients. Second, the average age of our patients was relatively high.

In conclusion, our study demonstrates that PPR does not associate with poorer oocyte quality or a decreased oocyte retrieval rate. Moreover, PPR is not related to a lower first FET LBR or cumulative LBR in the freeze-all cycle. In fact, our findings suggest that a lower progesterone level is associated with a lower cumulative LBR. These findings contradict the notion that PPR has adverse effects in the era of FET. Instead, our research highlights that PPR is associated with more retrieved oocytes with similar embryo quality and oocyte retrieval rates, resulting in a higher cumulative LBR even after accounting for age, AMH and gonadotropin dosage. Taken together, our findings underline that PPR is not a worrying parameter, as it may simply be due to higher ovarian responses and result in a higher cumulative LBR.

## Figures and Tables

**Table 1:** Baseline demographics, cycle characteristics, and embryology results of patients.

Progesterone Level (ng/mL)	<1.5 (*n* = 526)	≥1.5 (*n* = 149)	*p* Value
Age (years)	36.6 ± 4.0	35.8 ± 4.0	0.042
AMH (ng/mL)	3.3 ± 3.2	4.6 ± 3.2	<0.001 **
Total FSH dosage (IU)	3297.8 ± 1163.0	3123.3 ± 1015.8	0.090
Total LH dosage (IU)	1131.7 ± 676.9	991.1 ± 638.0	0.020 *
FSH/LH dosage ratio	4.0 ± 4.5	3.6 ± 2.3	0.119
Induction duration (days)	10.2 ± 1.7	10.5 ± 1.2	0.006 **
No. of ≥14 mm follicle	9.8 ± 6.4	14.0 ± 7.0	<0.001 **
Estradiol on day of hCG administration (pg/mL)	2895.1 ± 1775.8	4867.9 ± 2542.9	<0.001 **
No. of oocytes retrieved	13.9 ± 9.6	19.6 ± 10.2	<0.001 **
Oocyte retrieval rate	83.9% ± 24%	84.4% ± 25.9%	0.515
Rate of good embryos ^1^ (at day 3)	32.7% ± 25%	34.1% ± 21.5%	0.375
Blastocyte formation rate	55.8% ± 23.7%	59.6% ± 32.0%	0.370

* *p* < 0.05. ** *p* < 0.01. ^1^ Cleavage embryos were classified as good quality if they consisted of at least seven to eight cells graded 1 or 2 on day 3, based on the Veeck classification system.

**Table 2:** Univariate and multivariate logistic regression analysis for serum progesterone ≥ 1.5 ng/mL on the day of hCG administration.

	Univariate			Multivariable Model		
	OR	95%CI	*p* Value	OR	95%CI	*p* Value
Age (years)	0.94	(0.90–0.98)	0.003 **	1.03	(0.98–1.08)	0.321
AMH (ng/mL)	1.19	(1.14–1.25)	<0.001 **	1.02	(0.95–1.10)	0.582
Total dosage of FSH (IU)	1.00	(1.00–1.00)	0.158			
FSH/LH dosage ratio	0.99	(0.93–1.04)	0.623			
Induction duration (days)	1.27	(1.15–1.40)	<0.001 **	1.27	(1.14–1.42)	<0.001 **
No. of ≥14 mm follicle	1.14	(1.11–1.17)	<0.001 **			
Estradiol on day of hCG administration (pg/mL)	1.00	(1.00–1.00)	<0.001 **			
No. of oocytes retrieved	1.09	(1.07–1.10)	<0.001 **	1.08	(1.06–1.11)	<0.001 **
Rate of good embryos ^1^ (at day 3)	1.26	(0.55–2.90)	0.590			
Rate of good blastocyst ^2^ (at day 5/6)	2.00	(0.99–1.00)	0.808			
Oocyte retrieval rate	0.76	(0.70–1.35)	0.288			

** *p* < 0.01. ^1^ Cleavage embryos were classified as good quality if they consisted of at least seven to eight cells graded 1 or 2 on day 3, based on the Veeck classification system. ^2^ The grade according to the Gardner classification system of ‘3BA’ or greater was defined as good quality blastocyst.

**Table 3:** Baseline demographics, cycle characteristics, and embryology results of serum progesterone ≥ 1.5 ng/mL in different AMH groups.

AMH (ng/mL)	≤1.2			>1.2–≤3.36			>3.36		
Progesterone level (ng/mL)	<1.5	≥1.5	*p* value	<1.5	≥1.5	*p* value	<1.5	≥1.5	*p* value
Age (years)	39	39	0.623	37	37	0.362	35	35	0.428
AMH (ng/mL)	0.77	0.8	0.638	1.95	2.29	0.001 **	5.45	4.97	0.380
Total FSH dosage (IU)	3750	3975	0.293	3375	3525	0.045 **	2250	2700	0.007 **
FSH/LH dosage ratio	2.8	2.65	0.229	2.8	2.8	0.859	3.5	3.22	0.236
Induction duration (days)	10	11	0.006 **	10	11	0.002 **	10	11	<0.001 **
No. of ≥14 mm follicle	3	6	0.005 **	7	10	<0.001 **	13	16	<0.001 **
Estradiol on day of hCG administration (pg/mL)	1082	1900	0.006 **	1954	3514	<0.001 **	3569	5476	<0.001 **
No. of oocytes retrieved	4	8	0.002 **	9	14	<0.001 **	19	23	0.003 **
Blastocyte formation rate	33%	50%	0.137	50%	65%	0.002 **	58%	72%	0.025 **

** *p* < 0.01.

**Table 4:** Pregnancy outcomes of all patients and different groups of retrieved oocytes.

No. of Oocytes Retrieved	Progesterone on hCG Administration Day (ng/mL)	1st First Frozen Embryo Transfers Live Birth Rates	Cumulative Live Birth Rates with Freeze-All Strategy
All	<1.5	46.1%	59.3%
	≥1.5	51.1%	71.9%
	*p* value	0.209	0.002 **
≤5	<1.5	17.4%	20.4%
	≥1.5	16.7%	16.7%
	*p* value	0.965	0.825
6–19	<1.5	47.4%	59.7%
	≥1.5	53.2%	61.3%
	*p* value	0.256	0.856
≥20	<1.5	65.0%	88.3%
	≥1.5	65.1%	92.1%
	*p* value	0.934	0.432

** *p* < 0.01.

**Table 5:** Pregnancy outcomes of different progesterone quartiles in all patients.

Quartile	Progesterone on hCG Administration Day (ng/mL)	1st First Frozen Embryo Transfers Live Birth Rates	Cumulative Live Birth Rates with Freeze-All Strategy
First	≤0.5	26.4%	41.8%
Second	>0.5–0.72	47.1%	60.1%
Third	>0.72–1	47.7%	60%
Forth	>1	54.6%	71%
	*p* value	1st vs. 2nd: <0.001 ** 1st vs. 3rd: <0.001 ** 1st vs. 4th: <0.001 ** Other: non-significant	1st vs. 2nd: 0.004 ** 1st vs. 3rd: 0.005 ** 2nd vs. 4th: 0.024 ** 3rd vs. 4th: 0.025 ** 1st vs. 4th: <0.001 ** Other: non-significant

** *p* < 0.01.

**Table 6:** Univariate and multivariate logistic regression analysis for the cumulative live birth rate.

	Univariate			Multivariable Model		
	OR	95%CI	*p* Value	OR	95%CI	*p* Value
Age (years)	0.77	(0.75–0.80)	<0.001 **	0.81	(0.78–0.84)	<0.001 **
AMH (ng/mL)	1.50	(1.40–1.60)	<0.001 **	1.30	(1.20–1.40)	<0.001 **
Total dosage of FSH (IU)	1.00	(1.00–1.00)	<0.001 **	1.00	(1.00–1.00)	0.014 *
FSH/LH dosage ratio	1.20	(1.13–1.28)	<0.001 **			
Induction duration (days)	1.09	(1.02–1.16)	0.012 *			
No. of ≥ 14mm follicle	1.24	(1.20–1.27)	<0.001 **			
Estradiol on day of hCG administration (pg/mL)	1.00	(1.00–1.00)	<0.001 **			
No. of oocytes retrieved	1.17	(1.15–1.19)	<0.001 **			
Rate of good embryos ^a^ (at day 3)	2.35	(1.35–4.09)	0.003 **			
Rate of good blastocyst ^b^ (at day 5/6)	1.00	(1.00–1.00)	0.704			
Oocyte retrieval rate	1.65	(1.12–2.43)	0.011 *			
Premature progesterone rise	2.51	(1.86–3.37)	<0.001 **	1.56	(1.08–2.25)	0.017 *

* *p* < 0.05. ** *p* < 0.01. ^a^ Cleavage embryos were classified as good quality if they consisted of at least seven to eight cells graded 1 or 2 on day 3, based on the Veeck classification system. ^b^ The grade according to the Gardner classification system of ‘3BA’ or greater was defined as good quality blastocyst.

## Data Availability

Data will be available by contacting corresponding author.
